# Sensors Driven AI-Based Agriculture Recommendation Model for Assessing Land Suitability

**DOI:** 10.3390/s19173667

**Published:** 2019-08-23

**Authors:** Durai Raj Vincent, N Deepa, Dhivya Elavarasan, Kathiravan Srinivasan, Sajjad Hussain Chauhdary, Celestine Iwendi

**Affiliations:** 1 School of Information Technology and Engineering, Vellore Institute of Technology, Vellore 632 014, Tamil Nadu, India; 2 College of Computer Science and Engineering, University of Jeddah, Jeddah 21577, Saudi Arabia; 3 Department of Electronics, BCC of Central South University of Forestry and Technology, Changsha 410004, China

**Keywords:** smart agriculture, multi-layer perceptron, agricultural data, IoT in agriculture, land suitability using sensors, sensor data in agriculture

## Abstract

The world population is expected to grow by another two billion in 2050, according to the survey taken by the Food and Agriculture Organization, while the arable area is likely to grow only by 5%. Therefore, smart and efficient farming techniques are necessary to improve agriculture productivity. Agriculture land suitability assessment is one of the essential tools for agriculture development. Several new technologies and innovations are being implemented in agriculture as an alternative to collect and process farm information. The rapid development of wireless sensor networks has triggered the design of low-cost and small sensor devices with the Internet of Things (IoT) empowered as a feasible tool for automating and decision-making in the domain of agriculture. This research proposes an expert system by integrating sensor networks with Artificial Intelligence systems such as neural networks and Multi-Layer Perceptron (MLP) for the assessment of agriculture land suitability. This proposed system will help the farmers to assess the agriculture land for cultivation in terms of four decision classes, namely more suitable, suitable, moderately suitable, and unsuitable. This assessment is determined based on the input collected from the various sensor devices, which are used for training the system. The results obtained using MLP with four hidden layers is found to be effective for the multiclass classification system when compared to the other existing model. This trained model will be used for evaluating future assessments and classifying the land after every cultivation.

## 1. Introduction

Agriculture farming is considered as the base for human living because it is the primary source of food and income for most of the countries in the world. The economy of the country depends on agriculture production as it provides food, raw materials, employment, etc., to the people living in that country [1]. It has been observed in recent times that there is no significant development in crop production in the agriculture sector. Also, there is a rapid increase in the price of food because the production of the crop is not meeting demand [2]. One of the causes for the decrease in crop production is the farmers use of the traditional way of cultivation, which leads to less crop yield. The farmers new to the agriculture field have insufficient knowledge about the characteristics of soil for crop cultivation [3]. They are not conscious of the fact that agriculture land needs to be assessed before cultivation [4].

Land suitability analysis is a mandatory prerequisite for crop cultivation, which helps to obtain maximum production. In order to acquire the properties of the soil, farmers depend on soil testing labs, which are not sufficient enough to help them, and sometimes the data provided by the labs are inaccurate [5]. In order to get enough knowledge about cultivation, data need to be collected manually, which is very difficult for farmers. The solution is the replacement of traditional methods of data collection with Internet of Things (IoT)-based sensors [6]. Sensors play a significant role in collecting information about various factors such as soil, water, climate, etc., for agriculture development. With the help of data gathered from different sensors, land suitability analysis could be done, which would help farmers identify the current status of their agriculture land and improve their crop production. Several decision models have been developed so far to assist the farmers in taking a decision on crop cultivation to maximize their profit [7].

Ever-changing real-world conditions like monsoons to temperate are the dominant factors in affecting agriculture productivity. Soil parameters to weather prediction are essential in knowing the future yield [8]. The key factors that are influencing the crop yield are climatic conditions, soil productivity, and groundwater characteristics, as well as its availability. An extensive study on phenotyping is helping researchers to understand these factors [9]. One of the significant challenges involved in identifying the different land conditions is based on the influence of various soil parameters. Over the past few decades, soil testing has been practised, which is widely accepted by agriculture experts and farmers to determine the properties of soil for agriculture production [10]. The Agriculture Management Information System (AMIS) is mandatory to store the data acquired in various formats and exchanges the data for digital agriculture technology [11]. Currently, AMIS has enhanced its facilities by accumulating the latest technologies, such as the Internet and the Internet of Things (IoT).

Introduction of the usage of sensors actually reduces the cost and time involved in assessing the land suitability in the traditional manner [12]. The uses of sensors are plenty, and it is possible to use as many sensors in the field of agriculture. Soil sensors, water sensors, and biosensors are few that have been shown to have a significant role in measuring nature. These sensors contribute to the smart farming system, especially in the handling of appropriate irrigation systems to help farmers. The Radio Frequency Identification (RFID) technology is used for the detection of animals entering into the farmland. The Global Positioning System (GPS) position-based seeding recommendation is emerging as another benefit in the field of agriculture benefiting from the latest technological developments [13]. Some of the benefits it has achieved in recent days are improved efficiency, sustainable growth, as well as cost-effectiveness. It is considered to be the connection between the collection of physical devices through internet connection such as WiFi, Data cards, etc.

As a revolutionary change, the sensor-driven network has a considerable role in precision agriculture [14]. Timely forecast based on soil parameters will ensure proper irrigation systems. IoT has a significant role in handling the recorded data from sensors. General Purpose Input/Output facilitates various sensor inputs and the corresponding outputs in Raspberry Pi. Further, this could really help in hosting different sensors. Cloud computing is helping the IoT sensor networks to record the measured data through its sensors. The wireless medium is transferring the data to the cloud, which is used later for data analysis [15].

Data Analytics has brought impactful results for future predictions in almost every single application. Artificial intelligence-based model building is a challenging task as the model should replicate the observed parameters in the dataset. Parametric estimations are helpful in solving the problems in a futuristic way, and agriculture problems are among those [16]. Agriculture data analysis is done with different machine learning algorithms. Maximizing the outcome is the agenda for any machine learning model, and model assessment metrics are helpful in analyzing the results obtained. Appropriate training of the machine learning model will produce the results with utmost accuracy.

Neural network-based models are used widely, as they have the capability to improve the efficiency of the model over every iteration [17]. Classification problems are handled by various algorithms, such as support vector machines, decision tree algorithms, etc. Multiclass classifications are expertly handled by neural network algorithms [18]. When the model is trained with the appropriate number of inputs and outputs, it is expected to give better results compared to other machine learning models. The integration of IoT, along with machine learning models, are providing the farmer recommendation system with appropriate inputs [19].

In this work, an agriculture dataset has been obtained through various IoT sensor devices such as a pH sensor, soil moisture sensor, salinity sensor, and electromagnetic sensor. A sensor is a device which is used to detect and respond to some type of input obtained from the physical environment [20]. As it requires less labor and consumes less time, it is used in many real-time applications. As the Internet plays a mediatory role in various kinds of communication and data exchange, it is advisable to integrate the agriculture data with the cloud platform. The data acquired from the various IoT devices could be stored to the cloud platform [21].

The key contribution of this work is as follows

- The selection of the relevant attributes and the collection of data from the appropriate sources for better agriculture land suitability classification, and these data are heterogeneous in nature.
- Integration of different data collection and bringing it to a form suitable for future predictions.
- The noble initiation towards helping the farmers build a recommendation model, especially for the low economic regions, in a country like India.
- Generalization of the proposed model, which could be used for any land, as the current test inputs are sufficient for the classification of the land suitability with this developed model.
- The multiclass classification is another crucial contribution as the majority of the past work is based on binary classification, which includes two decision classes, namely suitable and unsuitable. These multiclass results are helpful for the farmers when compared to the binary classification, to take suitable action based on the outcome of the results.

This paper presents a model that will automate the process of analyzing various deciding factors in the assessment of present and future conditions for the better crop yield. The different classification will help decide well in advance how to proceed to start a new session. Besides, this would ensure minimal loss to the farmers, and the different sections presented here elucidate the model further.

## 2. Related Work

The integration of the Internet of Things into the agricultural system has led to the Internet of Agriculture of Things along with advanced computing techniques. The researchers apply this to obtain maximum benefit and to also improve the production of agriculture, artificial intelligence, and IoT [22]. The agriculture domain is experiencing new evolution and revolution motivated by sensors, IoT, big data, and cloud technology [23]. The IoT based on a smart agriculture system was developed using deep reinforcement learning integrated with a cloud environment, in which four layers were included, namely data collection, edge computing, data transmission, and cloud computing [24]. IoT has been integrated with agriculture systems to yield maximum profit, and the applications of IoT in agriculture have been categorized into restricted environment planning, open farm planting, livestock farm monitoring, and aquaculture development [25].

A new proposal was presented for agriculture applications by investigating various integrated platforms, which include cloud computing, IoT, and data mining techniques [26]. A scalable network-based architecture was proposed to monitor and regulate agriculture farms in rural areas with the help of IoT-based WiFi with long-distance network and fog computing [27]. The development of IoT in agriculture data analytics has transformed from a specific crop to any kind of crop. Also, the system is able to support a various number of applications from controlling and supervising the agriculture crops to promoting the products in the market [28]. An irrigation monitoring and control system was developed using a fuzzy approach to generate a moisture content distribution map of soil by applying an interpolation technique.

The IoT-based Wireless Sensor Network (WSN) was used to generate a distribution map with the measurements of soil moisture content taken at nine places of the selected farmland [29]. An IoT-based system was developed to water agriculture crops using a wireless sensor network, and a web application was also designed to control the information about the crop and field [30]. Several machine learning algorithms have been used so far for prediction and forecasting. A new forecasting model was designed by combining the gravitational search algorithm with Multilayer Perceptron (MLP) to forecast the level of water in Winnipesaukee and Cypress Lakes in the USA [31]. A neural network-based model was proposed using MLP to predict the bid price of the keyword in a particular search engine ranking [32]. A hybrid model was proposed by combining the firefly algorithm and MLP to predict the salinity value of soil with the help of remote sensing data [33]. An algorithm was proposed for the identification of weed diseases using MLP and automatic relevance determination. Three multi-criteria yield models were proposed using neural networks with MLP for the prediction of winter rapeseed [34].

An IoT-based hydroponic intelligent system was developed using deep neural networks to control the actions in the hydroponic environment characterized by multiple parameters gathered using IoT sensors [35]. An IoT-based smart decision model was developed to help the farmers to get information such as fertilizer requirements, crop analysis, and market requirements. IoT-based sensors were used to obtain the data related to farming, and it is stored in a cloud framework where information is processed and sent to farmers’ mobile devices by applying some data mining techniques [36]. An Intelligent model was developed for the diagnosis of faults in the aquaculture ponds located in remote places through IoT sensors. A fuzzy neural network algorithm was applied for fault detection, and the model detects many types of faults in aquaculture ponds [37]. An IoT-based prototype was designed for monitoring the water level in sources like borewells, water tanks, etc. This prototype could be used to avoid damage in pump motors when the water level goes beyond the threshold level [38].

A predictive platform for precision agriculture was presented using IoT devices to improve crop productivity at low-cost. Deep learning algorithms were used to predict the diseases in plants which were hidden in the images of the leaves, and they were classified with maximum accuracy [39]. An intelligent system was developed to predict the fruit-melon image of skin lesions and alert for new planting environments using the convolutional neural network and deep learning rule. The image is obtained by using an infrared video sensor, and the system has obtained a satisfactory accuracy of 97.5%. A modern automatic agriculture monitoring system was developed using the ZigBee Wireless Sensor Network and deep learning algorithms. Wireless sensor networks are also used to monitor the temperature of soil and humidity. Also, the system classifies the moisture content in the soil.

A decision tree-based model was proposed for optimizing water and energy consumption [40]. Further, this was accomplished with the assistance of the Internet-of-things. Moreover, to help the farmers in saving the precious water resource, which is declining day by day, several soil parameters were considered. Besides, for measuring the water requirement, and factors such as soil moisture content and soil temperature were utilized for making a suitable decisions concerning water release. The cloud-based IoT sensors are helpful in this work to control the overall setup. This in turn also saves the energy.

The below section presents the proposed system which is a recommendation model for using artificial intelligence and sensors.

## 3. Proposed System—Sensors Driven Artificial Intelligence (AI) - Based Agriculture Recommendation Model

**Figure 1:**
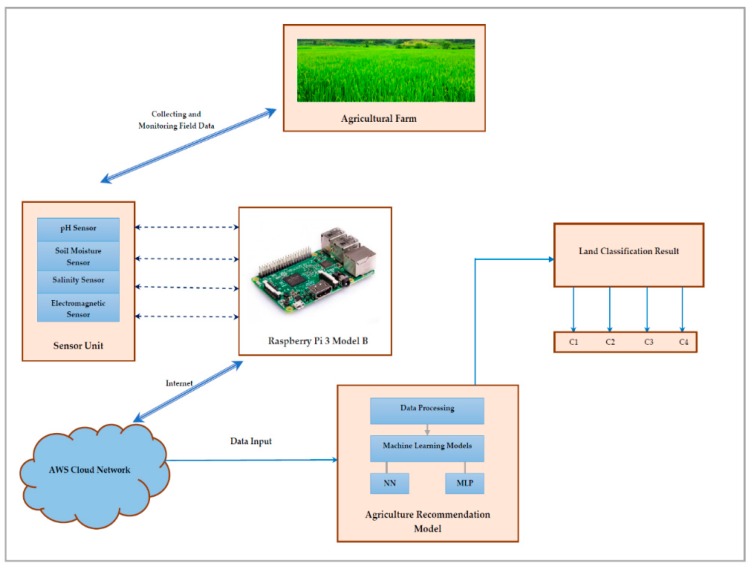
Architecture diagram of the proposed model.

The sensor network-based proposed model is discussed in this section. The architecture diagram of the sensor-based data management is shown in Figure 1. Sensor-based data collection requires three main steps: data acquisition, data communication, and data processing. For collecting the various parameter values concerning the properties of soil suitable for agriculture development, various sensors are used [41]. The data acquisition is made using various sensor devices such as the pH sensor, soil moisture sensor, salinity sensor, and an electromagnetic sensor.

The raspberry Pi 3 system is used here to handle inputs from multiple sensors, and the data are sent to a cloud for storage, since it has the most powerful CPU comparatively, as well as the IEEE 802.11 wireless standard. A wi-fi facility is also available and is used to transfer the data from the remote agriculture land. For the better handling of data, the data is sent further to the cloud with the help of the internet. The cloud facility used here is Amazon Web Serive (AWS), and the stored data is used for machine learning for the purpose of analysis. The data is accessed on the local machine through a cloud facility. The algorithm is developed in the machine and it is tested on the collected data to verify the accuracy of the results obtained. The Raspberry Pi controller is used to collect the data from various sensors for a sampling period of one day. Then the average values of various sensors are moved to the AWS Cloud Network with the help of the Internet. From the AWS, the data is accessed by the proposed model, which is developed in the system for normalization and training purposes. Thus, the data obtained from various sensors for six months are considered for the development of the sensor-driven AI model.

The various parameters used to find agriculture land suitability analysis in this work are soil texture, granular fragments (percentage of sand particles in the soil), structure of the soil, compact and cementation, cnternal drainage, available water content, porousness, organic matter, cation exchange capacity, degree saturation, pH value, salinity, and carbonates. Moreover, this is the collected information which is essential to make proper classification of land suitability. These inputs are collected from sensors, as well as from the anonymous farmers and agriculture experts. Their collective responses and inputs helped to construct the required dataset.

Electromagnetic sensors have been used to obtain the measurements for soil texture, internal drainage, available water content, organic matter, cation exchange capacity, carbonates, and degree of saturation. pH sensors are used to obtain the pH value, and salinity sensors are used to get the salinity value. The porousness value is obtained based on the measurement of the soil moisture sensor and granularity of the soil is obtained using the measurement acquired from the moisture content of the soil. The values of the structure of the soil and the compact and cementation are obtained from the agriculture experts.

The type of dataset acquired from the sensors are numerical values. Based on the values obtained from the sensor, suitability range values (obtained from experts) are assigned to the parameters considered for the land suitability model. For example, the pH value ranges from 7.2–6.7 for favorable land conditions, 6.6–5.5 for less favorable land conditions, 5.4–4.5 for unfavorable land conditions, and less than 4.5 for the very unfavorable land condition. The salinity value for the favorable land condition is <2, 2–6 for less favorable land conditions, 6–12 for unfavorable land conditions, and greater than 12 for the very unfavorable condition. The sampling period of data acquisition is one day. The average of the one-day data obtained from various sensors is stored in the cloud network as part of the preprocessing of the data. As the data may contain missing and noisy values, the mean of the data is considered. Since the data contain different units of measurement (categorical, numerical), normalization is done before applying the proposed model.

Figure 1 states the architectural diagram of the proposed model. The steps involved in this process are shown in this figure. The data is collected from the farmland using sensors, and is then transferred and stored with the help of the Raspberry Pi system in the cloud. The stored data is used here to build the artificial intelligence recommendation model shown in the figure. The constructed model is expected to classify the results into four different categories. Amazon Web Service (AWS) is used here for the storage of the data.

The learning model construction is explained in the algorithm part given below. The built model will be assessed with inputs received after every cultivation period, and accordingly, assessment results will be provided. Different classes are considered here for land suitability assessment as most suitable (class 1), suitable (class 2), moderately suitable (class 3), and unsuitable (class 4).

The Algorithm 1 presented here explains the steps involved in the processing the collected data from the different sensors. Since the size of the data is quite high in size, it is necessary to handle the data with the algorithm that is capable of handling the expected size of the data. That is the reason why a neural network approach is considered here for processing the data. The algorithm presented here is further explained below.

**Algorithm 1** MLP Algorithm for Machine Learning Model1: Initialize the weights (w) to small random numbers, Bias (b) and Learning rate (α)2: While (stopping criteria not met) do3:  For each training pattern $\left(x_{i},y_{i}\right)$ do4:  Process the input forward:5:  Activate each input: $x_{i}=s_{i}$, i∈1 to n
6:  Net input: $y_{i}=b+\sum_{i=1}^{n}x_{i}.w_{i}$
7:  where b = bias, n = number of neurons, y—actual output, t—target output8:  Final output with the following activation function:9:  
$f_{yin}=\left\{\begin{array}{c} \begin{array}{c} 1\;if\;y_{in\;}>\;\theta \\ 0\;if-\theta \leq \;y_{in\;}\leq \;\theta \end{array}\\ -1\;if\;y_{in\;}<\;-\theta \end{array}\right\}$
10: Adjust weight and bias:11:     
if y≠t,
12:     
$w_{i}\left(new\right)=w_{i}\left(old\right)+\propto t_{xi}$
13:     
b(new)=b(old)+ ∝t
14:  
if y=t,
15:    
$w_{i}\left(new\right)=w_{i}\left(old\right)$
16:    
b(new)=b(old)
17:  Calculating the average gradient:18:  Gradient calculation of each error with respect to $w_{i}$
19:  
$E\left(w\right)=\frac{1}{2}\sum_{i}\sum_{k\in y}{\left(t_{i}{}^{k}-y_{i}{}^{k}\right)}^{2}$
20:  where $t_{i}{}^{k},y_{i}{}^{k}$ are the target and the actual output at the ith input instance21:  end22: Calculation proceeds to the hidden layer23: end

Artificial Intelligence Model building is given in the algorithm. Processing of the neural networks relies upon the network topology, adjustments of weights, and activation functions. The network topology defines the network arrangement together with its connecting links and nodes. In neural networks, learning refers to the process of modifying weights of neural connections among the neurons of the specific network. Activation functions are additional efforts applied over the input to achieve the exact output. In the proposed work, a supervised learning-based neural network is adopted where the learning process is dependent. The input vector is offered to the network model and obtains an output vector, which is then compared to the desired or target vector. An error signal is developed in case of a variation existing between the actual and the desired output vector. Depending on the error signal the weights are adapted until the desired and the actual output matches. Neural networks can be precisely defined as complex adaptive architecture, which can transform its internal structure depending on the information passing through them. The following section explains the data collection and further processes.

## 4. Implementation of a Proposed Sensor Network-Based Model

### 4.1. Description of the Dataset and Study Area

The data used for this experimentation are collected from the different villages of the Vellore and Tiruvannamalai district in the state of Tamil Nadu, India. With expert opinions, we have shortlisted the various parameters that are really influence the crop yield. This collection of the dataset includes a combination of distinctive climate, soil, and groundwater properties. Four decision classes are considered here for land suitability assessment, namely most suitable (class 1), suitable (class 2), moderately suitable (class 3), and unsuitable (class 4).

### 4.2. Data Preparation

It is essential to prepare the information astutely before training the neural networks (NN) and multilayer perceptron (MLP) models. The present real-world data obtained from sensors are distributed non-uniformly, and henceforth, th data cannot be utilized legitimately amid training and testing of the NN and MLP models. In this way, the input features are normalized, and categorical variables are converted to numeric data through the data label encoder for efficient processing. The standardized dataset is then sub-divided into training and independent test sets in the ratio of 75:25. The results obtained are observed in the perspective of multiclass classification, as well as for the individual class basis. The process of training and testing is repeated for a variable number of iterations starting from 30, 50, and so on, till 100 until the convergence optimization is met, and also for a variable number of neurons and hidden processing layers for each model.

### 4.3. Performance Measures

The performance parameters which are assessed for multiclass classification are characterized as follows. For a class ‘C’, the classifier efficiency and performance could be assessed with true positive (TP), true negative (TN), false positive (FP), and false negative (FN) that can be determined from the counts of testing samples belonging to ‘C’ [42]. Various performance metrics appropriate for assessing NN and MLP for multiclass classification problems are calculated [42]. Classification accuracy scores could be used to determine the accuracy of the model by defining the number of correct predictions made from all the predictions. Also, this enables us to determine the per-class effectiveness of the model and is calculated as,

(1) $$\mathrm{Accuracy}=\frac{\mathrm{TP}+\mathrm{TN}}{\mathrm{TP}+\mathrm{TN}+\mathrm{FP}+\mathrm{FN}}$$

The performance metrics such as precision, recall, F1 score, etc., used for binary classification needs to be generalized for the multiclass classification setting. To meet the appropriate needs, we calculate the micro-average and the macro average. In micro-averaging, the performance is evaluated, i.e., the precision, recall, and the F1 score are obtained from the individual TP, TN, FP, and FN of the n-class model [42]. In macro-averaging, the mean of the performance of each individual class is determined, providing equal weight to each of the classification labels. Precision is the ratio of the predicted positive events that are actually positive.

(2) $${\;\mathrm{Precision}}_{\mathrm{micro}}=\;\frac{\sum_{i=1}^{n}{\mathrm{TP}}_{i}}{\sum_{i=1}^{n}\left({\mathrm{FP}}_{i}+{\mathrm{TP}}_{i}\right)}$$

(3) $${\;\mathrm{Precision}\;}_{\mathrm{macro}}=\;\frac{\sum_{i=1}^{m}\frac{{\mathrm{TP}}_{i}}{\left({\mathrm{FP}}_{i}+{\mathrm{TP}}_{i}\right)}}{n}$$

Recall, also known as sensitivity, is the ratio of the positive events that are predicted correctly.

(4) $${\mathrm{Recall}}_{\mathrm{micro}}=\;\frac{\sum_{i=1}^{n}{\mathrm{TP}}_{i}}{\sum_{i=1}^{n}\left({\mathrm{FN}}_{i}+{\mathrm{TP}}_{i}\right)}$$

(5) $${\mathrm{Recall}\;}_{\mathrm{macro}}=\;\frac{\sum_{i=1}^{m}\frac{{\mathrm{TP}}_{i}}{\left({\mathrm{FN}}_{i}+{\mathrm{TP}}_{i}\right)}}{n}$$

F1 score is the harmonic mean of the recall and the precision. The higher the F1 score, the better the model [27].

(6) $$F1{\;\mathrm{score}}_{\mathrm{micro}}=\frac{2\times {\mathrm{Precision}}_{\mathrm{micro}}\times {\mathrm{Recall}}_{\mathrm{micro}}}{{\mathrm{Precision}}_{\mathrm{micro}}+{\mathrm{Recall}}_{\mathrm{micro}}}$$

(7) $$F1{\;\mathrm{score}}_{\mathrm{macro}}=\frac{2\;\times {\mathrm{Precision}}_{\mathrm{macro}}\times {\mathrm{Recall}}_{\mathrm{macro}}}{{\mathrm{Precision}}_{\mathrm{macro}}+{\mathrm{Recall}}_{\mathrm{macro}}}$$

**Figure 2:**
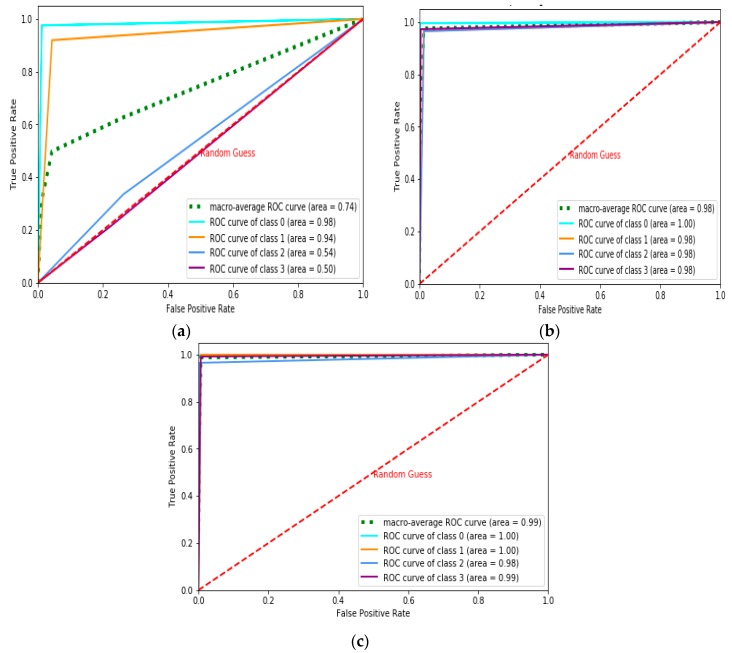
Multiclass Area Under Curve (AUC)-Receiver Operating Characteristics (ROC) curve for Neural Network (NN) model for: (**a**) $N_{h}$ = 30, (**b**) $N_{h}$ = 50, (**c**) $N_{h}$ = 80. **(**$N_{h}-\mathrm{Number}\;\mathrm{of}\;\mathrm{hidden}\;\mathrm{neurons})$

**Figure 3:**
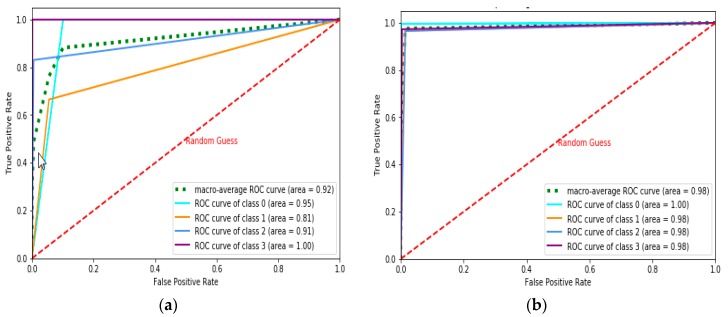

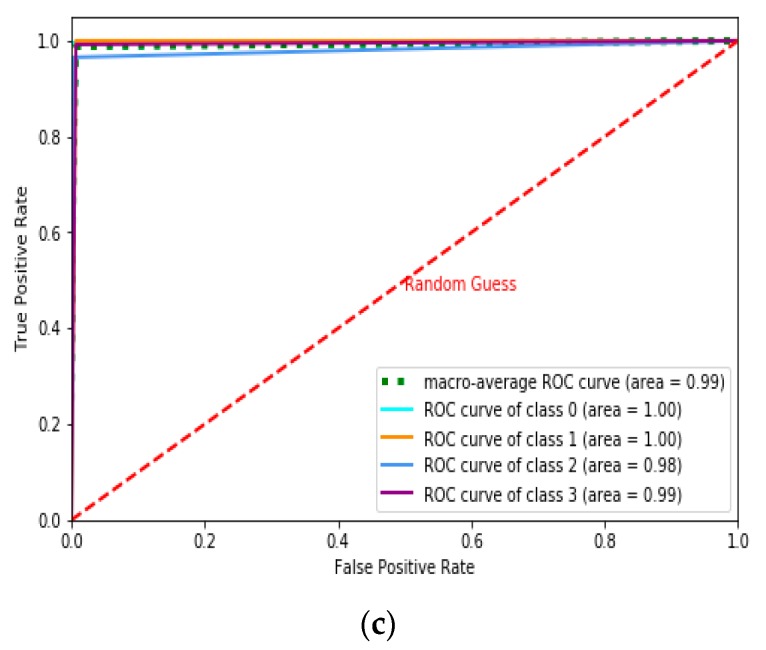
Multiclass AUC-ROC curve for MultiLayer Perceptron (MLP) model with three hidden layers for: (**a**) $N_{h}$ = 30, (**b**) $N_{h}$ = 50, (**c**) $N_{h}$ = 80.

**Figure 4:**
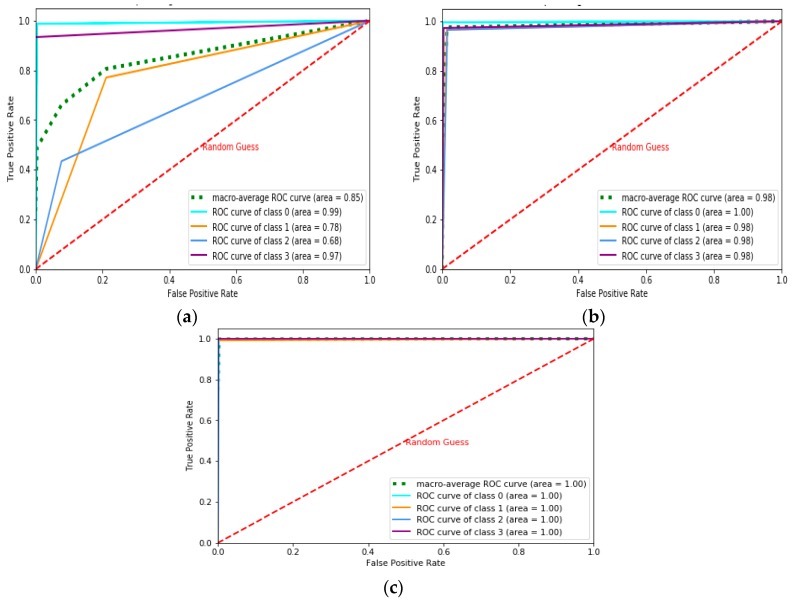
Multiclass AUC-ROC curve for MLP model with four hidden layers for: (**a**) $N_{h}$ = 30, (**b**) $N_{h}$ = 50, (**c**) $N_{h}$ = 80.

The Receiver Operating Characteristics (ROC)-Area Under the Curve (AUC) curve is the performance measurement for classification problems under various threshold settings. ROC represents the probability curve, and AUC is the degree or measure of separability distinguishing the classes. The AUC-ROC score is represented in Table 2 under various iterations for the NN and MLP learning models. Figure 2, Figure 3 and Figure 4 represent the AUC-ROC curve for the multiclass classification problem under various iterations for the learning models. The AUC-ROC curve indicates how well the probabilities of the positive class are separated from the negative class.

Besides the above-mentioned micro and macro-measures, the errors—Mean squared error (MSE), and root mean squared error (RMSE)—are also determined during the resulting process. Nevertheless, it is prudent to mention that the stochastic gradient descent does not require MSE and RMSE during training, instead it needs the error term between the random sample and its prediction for refurbishing the weights of the neural networks. MSE and RMSE for determining the average error of convergence of the model could be calculated as follows.

(8) $$\mathrm{MSE}=\frac{1}{N}\overset{N}{\underset{i=1}{\sum }}{\left(y_{i}-y_{i}^{\prime }\right)}^{2}$$

Here N is the total number of data samples, $y_{i}$ is the target or the hypothesis for ith data instance and $y_{i}^{\prime }$ is the outcome or the output of the ith data instance of the learning model.

## 5. Experimental Results

The proposed sensor-based AI model evaluates the experimental agriculture land based on the considered attributes for cultivating the crop, which is expected to give better yield. The agriculture data obtained from sensors consists of 1000 alternatives with 14 attributes. Out of 1000 data instances, 750 data instances are used for training the model, and the remaining 250 are used for testing the model. The test dataset has been collected separately after every crop yield in a span of one year. The proposed model classifies the experimental dataset into four decision classes, namely most suitable (class 1), suitable (class 2), moderately suitable (class 3), and unsuitable (class 4). The agriculture site belongs to class 1, and class 2 could be used for crop cultivation in the present form, whereas the agriculture farm belongs to class 3 needs to be further processed by applying the appropriate manure before cultivating the crop, and the land that comes under class 4 cannot be used for cultivation. The collected agriculture dataset here is applied to the MLP algorithm for training purpose. The performance of the MLP-based multiclass classification model is compared with the results obtained from neural networks.

The number of hidden layers and the number of neurons in each hidden layer are one of the most significant architectural parameters which legitimately impacts the execution of MLP and NN during the training and preparation of the information for setting up a learning base. In any case, determining these parameters prior has been an unsolved problem in ML research. In the proposed work for NN, the experiments were performed with the number of hidden neurons ($N_{h}$) to be set as 30, 50, and 80 and the results were noted. For MLP, the experiment is repeated for three and four numbers of hidden layers and the neurons in each layer were 30, 50, and 80. All the results have been epitomized and defined in tables for varying the $N_{h}$ values. Table 1 and Table 2 defines the results obtained for each class separately using NN and MLP with $N_{h}$ = 30. Likewise, Table 3 and Table 4 defines the results obtained for $N_{h}$ = 50 and $N_{h}$ = 80. On the other hand, Table 4 describes the results attained by the NN and MLP based on multiclass classification with $N_{h}$ = 30. Likewise, Table 5 and Table 6 illustrates the multiclass classification results obtained for $N_{h}$ = 50 and $N_{h}$ = 80 accordingly. It needs to be noticed that all the results that appeared in these three tables are averaged and equated over ten independent simulations for each of the learning models.

## 6. Result Analysis

The obtained classification results, which are delineated in Table 2, Table 3, Table 4, Table 5 and Table 6, are basically dependent on different performance parameters. The proposed work compiles various performance parameters for the evaluation and assessment of NN and MLP for the multiclass classification dataset. The training performance for the NN for the proposed dataset is found to be varying for different values of $N_{h}$ such as $N_{h}$ = 30, $N_{h}$ = 50, and $N_{h}$ = 80. The performance measures of the NN model are found to improve as the number of neurons in the hidden layer $N_{h}$ increases. With the increase in $N_{h}$, the NN is capable of predicting the test data hypothesis with improved accuracy. Moreover, the MSE and the RMSE are found to be very low and decrease accordingly in all three cases. Further, this results in better convergence of error, leading to possibly proper learning of features. The performance parameters, like the accuracy, precision, and others mentioned, seems to follow a similar manner.

Hence, it could be presumed that predictive accuracy could be a good measure for this dataset. The performance results obtained using the MLP with three hidden layers are found to be much better than that of NN. Similar to the NN model, this MLP model shows improved performance results with the increasing number of $N_{h}$. However, since the number of hidden layers has increased the architectural complexity, the convergence optimization is achieved with less number of iterations when compared to the NN.

The accuracy and other performance measures are found to improve accordingly with an increase in the $N_{h}$. On observing the performance measures of MLP with four hidden layers, the model individually is found to provide better results with improved performance with an increase in $N_{h}$. However, when observed for the test case of $N_{h}$ = 30, MLP with three hidden layers is found to produce better results than the MLP with four hidden layers. Further, this may be due to the increase in architectural complexity with an increased number of hidden layers. Also. if the $N_{h}$ is set to as high as 100, there might be a high risk of over-fitting the training data which would lead to poor performance than those obtained with $N_{h}$ values of 50 and 80. Besides, provided the current experimental setup, one could likewise accomplish slightly different results due to the underlying fact that the biases and initial weights of the neural networks are determined randomly. If an appropriate weight set is fixed initially, one could obtain superior results for similar settings. This assertion could be endorsed by the fact that the gradient descent may not generally ensure a near-ideal weight set towards the end of the neural network training process.

According to the results obtained inTable 6, the performance of MLP with four hidden layers is high compared to the performance of the Neural Network and the MLP with three hidden layer approaches used in some of the existing literature. Therefore, the proposed model assesses the given agriculture land and provides better results for sustainable agriculture development compared to other approaches. This model provides a reliable decision on the suitability level of the agriculture land in four different categories, helping agriculturists to assess their land appropriately. Thus, this proposed model could be used as a recommendation model for land suitability to improve the crop production for sustainable agriculture development.

## 7. Conclusions

Since agriculture is the backbone of any country, it is necessary to ensure its sustainable growth over the years. This work has presented a model that would be as accurate as 99%, which would be the most desirable. The data collected through various sensors, handled here with MLP with four hidden layers, has ensured better efficiency. A proper advisory system with precise instructions would always deliver better results. Thus, the accuracy score to precision score presented depicts the efficiency of this proposed approach, which will ensure appropriate classification. Multiclass classification in agriculture would further fine-tune the recommendation system to guide farmers appropriately. Rather than binary classification, this one would guide the farmers’ precisely. Thus, this approach would provide real-time data to ensure better crop yield productivity.

## Figures and Tables

**Table 1:** Performance of Neural Networks (NN) and MultiLayer Perceptron (MLP) with $N_{h}$ = 30 for each class averaged over ten independent runs.

Performance Measures	Performance of NN				Performance of MLP with Three Hidden Layers				Performance of MLP with Four Hidden Layers			
	Class 1	Class 2	Class 3	Class 4	Class 1	Class 2	Class 3	Class 4	Class 1	Class 2	Class 3	Class 4
Accuracy	0.86	0.88	0.85	0.83	0.93	0.96	0.94	0.945	0.91	0.93	0.924	0.912
${\mathrm{Recall}}_{\mathrm{micro}}$	0.85	0.87	0.862	0.884	0.951	0.94	0.92	0.938	0.92	0.914	0.93	0.911
${\mathrm{Recall}}_{\mathrm{macro}}$	0.20	0.215	0.223	0.218	0.23	0.22	0.23	0.217	0.22	0.229	0.21	0.23
${\mathrm{Precision}}_{\mathrm{micro}}$	0.875	0.864	0.881	0.87	0.951	0.96	0.95	0.959	0.92	0.926	0.931	0.93
${\mathrm{Precision}}_{\mathrm{macro}}$	0.22	0.204	0.213	0.221	0.239	0.24	0.23	0.248	0.22	0.236	0.221	0.23
${F1\;\mathrm{Score}}_{\mathrm{micro}}$	0.88	0.892	0.887	0.891	0.948	0.96	0.95	0.946	0.91	0.92	0.924	0.918
${F1\;\mathrm{Score}}_{\mathrm{macro}}$	0.21	0.224	0.217	0.23	0.219	0.23	0.22	0.235	0.22	0.232	0.221	0.23
AUC-ROC Score	0.86	0.88	0.88	0.89	0.97	0.95	0.97	0.969	0.95	0.952	0.96	0.952
MSE	0.235	0.241	0.24	0.237	0.047	0.04	0.04	0.042	0.08	0.087	0.091	0.09
RMSE	0.26	0.27	0.274	0.265	0.121	0.10	0.11	0.12	0.27	0.31	0.33	0.29

**Table 2:** Performance of NN and MLP with $N_{h}$ = 50 for each class averaged over ten independent runs.

Performance Measures	Performance of NN				Performance of MLP with Three Hidden Layers				Performance of MLP with Four Hidden Layers			
	Class 1	Class 2	Class 3	Class 4	Class 1	Class 2	Class 3	Class 4	Class 1	Class 2	Class 3	Class 4
Accuracy	0.97	0.982	0.971	0.969	0.98	0.99	0.98	0.987	0.99	0.99	0.998	0.99
${\mathrm{Recall}}_{\mathrm{micro}}$	0.98	0.975	0.981	0.974	0.97	0.98	0.98	0.976	0.9	0.98	0.99	0.987
${\mathrm{Recall}}_{\mathrm{macro}}$	0.23	0.24	0.248	0.25	0.24	0.25	0.24	0.247	0.24	0.25	0.25	0.25
${\mathrm{Precision}}_{\mathrm{micro}}$	0.981	0.97	0.984	0.975	0.97	0.98	0.97	0.979	0.98	0.99	0.994	0.99
${\mathrm{Precision}}_{\mathrm{macro}}$	0.25	0.257	0.249	0.25	0.25	0.24	0.25	0.242	0.25	0.249	0.25	0.25
${F1\;\mathrm{Score}}_{\mathrm{micro}}$	0.972	0.979	0.98	0.981	0.99	0.98	0.99	0.991	0.98	0.991	0.997	0.995
${F1\;\mathrm{Score}}_{\mathrm{macro}}$	0.25	0.242	0.239	0.24	0.249	0.25	0.25	0.24	0.25	0.249	0.24	0.25
AUC-ROC Score	0.99	0.987	0.982	0.993	0.99	0.98	0.99	0.99	0.99	0.987	0.99	0.98
MSE	0.018	0.02	0.014	0.01	0.001	0.00	0.00	0.001	0.00	0.001	0.001	0.001
RMSE	0.11	0.101	0.112	0.104	0.01	0.02	0.01	0.019	0.04	0.039	0.04	0.04

**Table 3:** Performance of NN and MLP with $N_{h}$ = 80 for each class averaged over ten independent runs.

Performance Measures	Performance of NN				Performance of MLP with Three Hidden Layers				Performance of MLP with Four Hidden Layers			
	Class 1	Class 2	Class 3	Class 4	Class 1	Class 2	Class 3	Class 4	Class 1	Class 2	Class 3	Class 4
Accuracy	0.98	0.99	0.987	0.99	0.99	0.98	0.99	0.985	0.99	0.99	0.987	0.99
${\mathrm{Recall}}_{\mathrm{micro}}$	0.99	0.995	0.993	0.984	0.994	0.99	0.99	0.992	0.98	0.99	0.996	0.989
${\mathrm{Recall}}_{\mathrm{macro}}$	0.25	0.245	0.249	0.25	0.25	0.25	0.24	0.248	0.25	0.249	0.237	0.25
${\mathrm{Precision}}_{\mathrm{micro}}$	0.98	0.986	0.99	0.992	0.99	0.99	0.98	0.991	0.99	0.986	0.992	0.99
${\mathrm{Precision}}_{\mathrm{macro}}$	0.25	0.248	0.24	0.25	0.25	0.25	0.25	0.24	0.25	0.251	0.25	0.248
${F1\;\mathrm{Score}}_{\mathrm{micro}}$	0.992	0.984	0.99	0.991	0.997	0.99	0.98	0.99	0.99	0.987	0.989	0.99
${F1\;\mathrm{Score}}_{\mathrm{macro}}$	0.25	0.247	0.25	0.249	0.25	0.24	0.24	0.25	0.24	0.248	0.25	0.25
AUC-ROC Score	0.99	0.97	0.99	0.985	0.997	0.99	0.99	0.987	0.99	0.97	0.985	0.99
MSE	0.01	0.03	0.01	0.01	0.001	0.00	0.09	0.001	0.00	0.00	0.01	0.001
RMSE	0.058	0.06	0.05	0.068	0.004	0.01	0.00	0.004	0.00	0.005	0.002	0.004

**Table 4:** Performance of NN and MLP with $N_{h}$ = 30 for multiclass classification averaged over ten independent runs.

Performance Measures	Performance of NN	Performance of MLP with Three Hidden Layers	Performance of MLP with Four Hidden Layers
Accuracy	0.89	0.959	0.926
${\mathrm{Recall}}_{\mathrm{micro}}$	0.897	0.96	0.92
${\mathrm{Recall}}_{\mathrm{macro}}$	0.22	0.24	0.23
${\mathrm{Precision}}_{\mathrm{micro}}$	0.88	0.963	0.93
${\mathrm{Precision}}_{\mathrm{macro}}$	0.21	0.241	0.23
${F1\;\mathrm{Score}}_{\mathrm{micro}}$	0.895	0.956	0.926
${F1\;\mathrm{Score}}_{\mathrm{macro}}$	0.223	0.239	0.231
Multiclass AUC-ROC Score	0.89	0.972	0.95
MSE	0.24	0.04	0.09
RMSE	0.275	0.116	0.3

**Table 5:** Performance of NN and MLP with $N_{h}$ = 50 for multiclass classification averaged over ten independent runs.

Performance Measures	Performance of NN	Performance of MLP with Three Hidden Layers	Performance of MLP with Four Hidden Layers
Accuracy	0.98	0.99	0.999
${\mathrm{Recall}}_{\mathrm{micro}}$	0.983	0.988	0.99
${\mathrm{Recall}}_{\mathrm{macro}}$	0.245	0.25	0.25
${\mathrm{Precision}}_{\mathrm{micro}}$	0.989	0.989	0.99
${\mathrm{Precision}}_{\mathrm{macro}}$	0.26	0.254	0.25
${F1\;\mathrm{Score}}_{\mathrm{micro}}$	0.986	0.992	0.99
${F1\;\mathrm{Score}}_{\mathrm{macro}}$	0.246	0.25	0.25
Multiclass AUC-ROC Score	0.991	0.998	0.998
MSE	0.016	0.001	0.001
RMSE	0.103	0.01	0.04

**Table 6:** Performance of NN and MLP with $N_{h}$ = 80 for multiclass classification averaged over ten independent runs.

Performance Measures	Performance of NN	Performance of MLP with Three Hidden Layers	Performance of MLP with Four Hidden Layers
Accuracy	0.99	0.998	0.999
${\mathrm{Recall}}_{\mathrm{micro}}$	0.993	0.995	0.998
${\mathrm{Recall}}_{\mathrm{macro}}$	0.248	0.25	0.25
${\mathrm{Precision}}_{\mathrm{micro}}$	0.99	0.99	0.99
${\mathrm{Precision}}_{\mathrm{macro}}$	0.247	0.25	0.25
${F1\;\mathrm{Score}}_{\mathrm{micro}}$	0.993	0.998	0.99
${F1\;\mathrm{Score}}_{\mathrm{macro}}$	0.25	0.249	0.25
Multiclass AUC-ROC Score	0.99	0.998	0.99
MSE	0.01	0.001	0.001
RMSE	0.06	0.004	0.003
